# Association of Neutrophil–Lymphocyte Ratio and the Presence of Neonatal Sepsis

**DOI:** 10.1155/2020/7650713

**Published:** 2020-12-02

**Authors:** Tiewei Li, Geng Dong, Min Zhang, Zhe Xu, Yidi Hu, Bo Xie, Yuewu Wang, Bangli Xu

**Affiliations:** ^1^Zhengzhou Key Laboratory of Children's Infection and Immunity, Children's Hospital Affiliated to Zhengzhou University, Henan Children's Hospital, Zhengzhou Children's Hospital, Zhengzhou, China; ^2^The Engineering Research Center for New Drug Screening, Inner Mongolia Medical University, Hohhot, China; ^3^Department of Neonatology, Children's Hospital Affiliated to Zhengzhou University, Henan Children's Hospital, Zhengzhou Children's Hospital, Zhengzhou, China

## Abstract

The neutrophil–lymphocyte ratio (NLR) is an emerging risk factor of sepsis that is receiving increasing attention. However, the relationship between NLR and the presence of sepsis in neonates is poorly studied. Here, we retrospectively recruited 1480 neonates and collected and analyzed relevant clinical and laboratory data. According to the International Pediatric Sepsis Consensus, 737 neonates were diagnosed with sepsis, and 555 neonates were suspected for having infection. Neonates with hyperbilirubinemia (*n* = 188) served as controls. Neonates with sepsis had significantly elevated neutrophil counts and NLR (*P* < 0.001). The proportion of neonates with sepsis increased significantly from 41.6% when NLR < 0.91 to 66.2% when NLR > 1.88 group (*P* < 0.001). Multiple logistic regression analysis showed that NLR was an independent risk factor for the presence of neonatal sepsis. Receiver operating characteristic (ROC) curve analysis showed that the optimal cut-off value NLR for predicting the presence of neonatal sepsis was 1.62 (area under curve (AUC) = 0.63, 95% CI 0.60–0.66, *P* < 0.001). In conclusion, our data suggest that elevated NLR levels are associated with a higher neonatal sepsis risk.

## 1. Introduction

Neonatal sepsis is one of the leading causes of morbidity and mortality in newborn infants [1] and is an important global public health issue [2]. Fleischmann-Struzek et al. reported that approximately 2202/100,000 livebirths develop neonatal sepsis, with mortality between 11% and 19% [3]. Early diagnosis and treatment of neonatal sepsis can help to prevent severe and life-threatening complications and subsequently reduce mortality. Clinical signs of neonatal sepsis include feeding intolerance, tachycardia, respiratory distress, pneumonia, and temperature instability, but these clinical symptoms often overlap with other noninfectious conditions making the diagnosis of neonatal sepsis difficult [1]. The gold standard for diagnosis of neonatal sepsis is blood culture [4], but blood cultures generally has a long waiting period, and the rate of positive cultures is low due to the small volume of blood used for inoculation and to contamination or antibiotics used before blood culture [5, 6]. It is clear that better predictors are needed to diagnose neonatal sepsis. Circulating blood biomarkers that may be useful in the early diagnosis of neonatal sepsis have been processed [7].

Sepsis is a systemic inflammatory response syndrome caused by infection, and inflammation plays an important role in the initiation and progression of sepsis. White blood cells and their subpopulations are vital in immune system defenses against pathogen infection. Many clinical studies have shown that neutrophil counts, lymphocyte counts, and the neutrophil to lymphocyte ratio (NLR) are predictors of sepsis [8–10]. NLR is considered to be more stable than absolute neutrophil or lymphocyte counts as both neutrophil and lymphocyte counts are included in the calculation [11]. NLR has attracted substantial attention due to its potential as a new risk factor for sepsis [12–14]. However, most studies on the relationship between NLR and sepsis are conducted in adult patients [8], and there have only been a few reports on the association between NLR and neonatal sepsis, all with a relatively small sample sizes [15–18]. Therefore, the aim of this study is to evaluate the relationship between NLR and neonatal sepsis in a relatively large neonatal population.

## 2. Materials and Methods

### 2.1. Study Design and Population

A hospital-based retrospective case-control study was conducted from January 2016 to December 2019 in Henan Children's Hospital. A total 1480 neonates were included in the study, and clinical and laboratory data were collected. Patients with the following conditions were excluded: (1) missing total and differential leukocyte counts and (2) other diseases, such as hematological system diseases, major congenital malformation, and cyanotic congenital heart disease. The study protocol complied with the Declaration of Helsinki and was approved by the hospital ethics review board. Written informed consent was obtained from all the participants.

### 2.2. Clinical Evaluation and Definition

The diagnosis of clinical neonatal infection and sepsis was made by two independent doctors according to the International Pediatric Sepsis Consensus [4]. Infection was defined as a suspected or proven infection caused by any pathogen or clinical sign associated with a high probability of infection, including abnormal temperature or leukocyte count, cough, white blood cells in a normally sterile body fluid, perforated viscus, chest radiograph consistent with pneumonia, petechial or purpuric rash, and purpura fulminans. Neonatal sepsis was defined as the presence of two or more systemic inflammatory response syndrome (SIRS) criteria owing to suspected or proven infection, one being an abnormal temperature or leukocyte count. The criteria for SIRS are as follows: (1) body temperature of more than 38.5°C or less than 36°C; (2) mean heart rate > 2 SD above normal for age in the absence of external stimuli, or unexplained persistent elevation for children < 1 year old, or mean heart rate < 10th percentile for age, or unexplained persistent depression over a 0.5 hr time period; (3) mean respiratory rate of more than 2 SD above normal for age or in the presence of mechanical ventilation; and (4) abnormal leukocyte count or >10% immature neutrophils. The detail information can be referred to the published International Pediatric Sepsis Consensus [4].

### 2.3. Laboratory Measurements

Blood samples were collected on admission to the hospital. WBC count and neutrophil and lymphocyte counts were measured by an automated blood cell counter (Sysmex Corporation, Kobe, Japan). The NLR was calculated by dividing the absolute neutrophil count by the lymphocyte count. High-sensitivity C-reactive protein (hsCRP) was measured using a latex-enhanced immunoturbidimetric assay (Ultrasensitive CRP kit, Upper Bio-Tech, Shanghai, China) on an UPPER analyzer (Upper Bio-Tech, Shanghai, China). hsCRP levels below 0.8 mg/L (measurement limits) were considered as 0.7 mg/L. Procalcitonin (PCT) levels were measured using an electrochemiluminescence assay (Elecsys® BRAHMS PCT kit, Roche Diagnostic, Rotkreuz, Switzerland) on a Cobas® 8000 modular analyzer (Roche Diagnostic, Rotkreuz, Switzerland). PCT levels above 100 ng/mL or below 0.02 ng/mL (measurement limits) were considered as 101 ng/mL and 0.01 ng/mL, respectively. The levels of serum total bilirubin (TBIL), aspartate aminotransferase (AST), alanine aminotransferase (ALT), total protein (TP), albumin (ALB), alanine aminotransferase (ALT), aspartate aminotransferase (AST), urea nitrogen (UREA), creatinine (CREA), and uric acid (UA) were measured using an automatic biochemistry analyzer (AU5800 Clinical Chemistry Analyzers, Beckman Coulter, California) and a conventional clinical analytical method.

### 2.4. Statistical Analysis

Statistical analysis was performed using SPSS 21.0 (SPSS Inc., Chicago, Illinois). The normality of variable data was tested prior to further statistical analysis. Normally distributed variables were expressed as the mean ± standard deviation (SD) and analyzed by independent *t*-tests or one-way ANOVA, as appropriate. Nonnormally distributed variables were presented as medians (interquartile range) and analyzed using the Mann–Whitney *U* test. Categorical variables were expressed as number and percentages (*n*, %) and assessed by chi-squared or Fisher exact tests. Analysis of correlations between two continuous variables was performed with a Pearson correlation test. Multivariate logistic regression analysis was performed to identify the independent risk factors for the presence of neonatal sepsis. The risk factors were prespecified, based on univariate *P* values below 0.05 and previously published literature. Receiver operating characteristic (ROC) curves were performed to evaluate the predictive value of NLR for the presence of neonatal sepsis. Youden's index was calculated (sensitivity + specificity − 1) to determine the optimal cut-off point. A two-sided *P* value below 0.05 was considered statistically significant.

## 3. Results

### 3.1. Baseline Clinical Characteristics of the Study Population

A total of 1480 neonates were enrolled in the study (average age: 8.0 (5.0, 14.7) days; 60.0% were males) and were divided into 3 groups based on whether they were diagnosed with infection or sepsis. 737 of the neonates were diagnosed with sepsis, and 555 neonates were suspected for having infections. 188 neonates with hyperbilirubinemia were served as controls. Baseline clinical and laboratory data for the three groups are summarized in Table 1.

**Figure 1:**
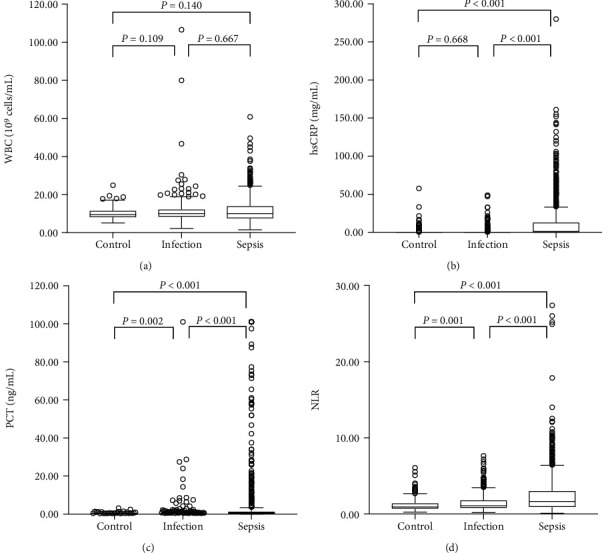
The levels of NLR and other inflammatory markers in control, infection, and sepsis group. (a) WBC had no significant difference among 3 groups. (b) hsCRP significantly increased in neonates with sepsis. (c, d) PCT and NLR showed a gradual increase among 3 groups.

Neonates with sepsis were older than the neonates with no infection and those with infection (10.0 (5.0, 17.0) vs. 7.0 (5.0, 12.0) and 7.0 (4.0, 12.0) days, *P* < 0.001). Clinical data showed that neonates with sepsis had a higher body temperature, respiratory rate, and heart rate than controls and neonates with infection (*P* < 0.001). Serum biochemical analysis showed that the levels of PCT, ALT, UREA, and UA were significantly higher in neonates with sepsis (*P* < 0.05), while serum TBIL, TP, and ALB levels were lower (*P* < 0.001). No differences were found in the levels of AST and CREA among the three groups. In addition, while inflammatory biomarkers such as hsCRP, neutrophil count, lymphocyte count, and NLR were significantly different between the three groups (*P* < 0.001), only PCT, neutrophil count, and NLR showed a gradual increase (Figure 1).

### 3.2. Association of NLR with the Presence of Neonatal Sepsis

To investigate the association of the NLR levels with the presence of neonatal sepsis, we classified the study participants into three groups according to NLR tertiles. As shown in Table 2, neonates in tertile 3 were younger (*P* < 0.001) and had a higher body temperature and respiratory rate (*P* =0.001). In addition, the levels of PCT, hsCRP, UREA, CREA, and UA were significantly higher in tertile 3 (*P* <0.001), while TBIL, TP, and ALB levels were lower in tertile 3. Further analysis showed that the prevalence of neonatal sepsis increased significantly from 41.6% in tertile 1 to 66.2% in tertile 3 group (*P* < 0.001), and neonates with infection were more likely to be in tertile 1 and tertile 2 (*P* < 0.001).

### 3.3. Correlation between NLR and Clinical Parameters

To further explore the relationship between the NLR and clinical parameters, we performed Spearman correlation analysis. As shown in Table 3, NLR was negatively correlated with age (*r* = −0.378, *P* < 0.001), TBIL (*r* = −0.111, *P* < 0.001), TP (*r* = −0.187, *P* < 0.001), and ALB (*r* = −0.249, *P* < 0.001). Meanwhile, the NLR positively correlated with PCT (*r* = 0.531, *P* < 0.001), hsCRP (*r* = 0.255, *P* < 0.001), UREA (*r* = 0.251, *P* < 0.001), CREA (*r* = 0.294, *P* < 0.001), and UA (*r* = 0.232, *P* < 0.001). In addition, there was no significant correlation between NLR and heart rate, AST, or ALT (all *P* > 0.05).

### 3.4. Independence of NLR Levels in Predicting Neonatal Sepsis

Univariate and multivariable binary logistic regression analysis was performed to assess the value of NLR in predicting the presence of neonatal sepsis. Variables in univariate analysis with *P* < 0.05 were included in a model for multivariate analysis, including age, heart rate, respiratory rate, weight, TP, ALB, CREA, TBIL, PCT, and hsCRP. Multivariate analysis showed that NLR was an independent predictor of the presence of neonatal sepsis (odds ratio (OR) = 1.445, 95% CI 1.301-1.604, *P* < 0.001) (Table 4). Further analysis showed that NLR was also independent of PCT and hsCRP (OR = 1.331, 95% CI 1.190-1.604, *P* < 0.001). In addition, our multivariate logistic regression models also confirmed that NLR tertiles were also independently associated with an increased prevalence of neonatal sepsis.

### 3.5. Receiver Operating Characteristic Curve Analysis

**Figure 2:**
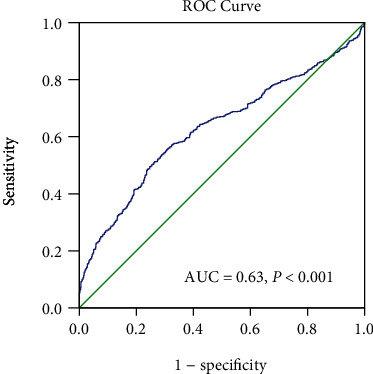
Receiver operating characteristic (ROC) curves of NLR to predict the presence of neonatal sepsis.

**Figure 3:**
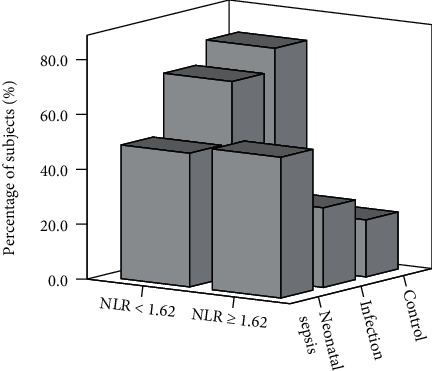
The distribution of subjects in high (≥ 1.62) or low (< 1.62) NLR groups.

Receiver operating characteristic (ROC) curve analysis was performed to evaluate the utility of NLR in predicting the presence of neonatal sepsis. Area under the ROC curve (AUC) indicated a well discriminatory power of NLR (AUC = 0.63, 95% CI 0.60–0.66, *P* < 0.001). Optimal cut-off value of NLR for predicting the presence of neonatal sepsis was 1.62, with a sensitivity of 51% and specificity of 75% (Figure 2). According to the cut-off value, we divided the subjects into the two groups (high NLR group ≥ 1.62 and low NLR group < 1.62). As shown in Figure 3, the high NLR group tended to have higher prevalence of neonatal sepsis and low percentage of no stenosis compared to the low NLR group.

## 4. Discussion

Neonatal sepsis is a serious life-threatening disease in neonates. According to the report by Li et al. [19] in 2015, 2.76 million children worldwide died in the neonatal period, and neonatal sepsis accounted for 15.2% of these deaths. Early diagnosis of neonatal sepsis is important for early treatment interventions, which in turn can prevent the incidence of serious life-threatening complications and death. Currently, the criteria for diagnosing of neonatal sepsis are mainly based on clinical signs, but these clinical signs are nonspecific [20]. Blood culture is the gold standard but takes up to 48 h to obtain results. In addition, blood culture is insensitive and can be affected by multiple factors, such as maternal antimicrobial treatment, inadequate volume of blood, and contamination [21]. Identifying rapid, sensitive, and specific new biomarkers is therefore critical.

Neonatal sepsis is a systemic inflammatory response syndrome caused by the invasion of specific or suspected pathogens into the blood and the continuous reproduction of toxins. It is accompanied by pathological inflammation and organ system dysfunction [1]. Neutrophils are an essential arm of the innate immune response during sepsis and release inflammatory cytokines, chemokines, and regulatory cytokines. Neutrophils can also engulf invading pathogens and kill them via a range of antimicrobial peptides, proteases, and oxidants [22]. In recent years, the discovery of neutrophil extracellular traps (NETs) has uncovered a new weapon in the immune system defenses against pathogen infection [23–25]. However, excessive expression of inflammatory cytokines and NET formation contributes to excessive inflammation and tissue damage [26–28]. Lymphocytes are also involved in the immune response against infections by bacteria and viruses. During pathogen infection, antigen-presenting cells recognized microbial antigens and presented it to T cells. Subsequently, CD4^+^ T cells secrete cytokines helping phagocytotic cells to kill intracellular bacteria [29]. However, during sepsis, the number of lymphocytes declines significantly due to apoptosis. This decline is considered an important contributing factor to the immunosuppressive state that makes patients vulnerable to new infections [30, 31].

Total and differential leukocyte counts represented cheap, widely available indicators of the inflammatory response. NLR reflects changes in neutrophil and lymphocyte counts. Many studies have demonstrated that NLR represents a reliable inflammatory marker and prognostic index in a variety of medical conditions, including ischemic stroke [32, 33], cerebral hemorrhage [34, 35], major adverse cardiac events [36–39], and solid tumors [40, 41]. Recently, the NLR has attracted substantial attention as a new risk factor with potential for use in the diagnosis of sepsis. Sepsis could give rise to elevated neutrophil counts and decreased lymphocyte counts resulting from the infection of pathogenic microorganisms, indicating that sepsis patients might have a higher NLR level [10, 42–44]. Numerous epidemiological investigations and meta-analysis studies have demonstrated that NLR may be a helpful predictor of sepsis, and patients with higher NLR may have a higher risk of unfavorable prognosis [12, 45]. However, most studies on the relationship between NLR and sepsis have been conducted in adults. There are only a few published studies (based on small sample sizes, *n* < 150) showing that NLR is positively correlated with neonatal sepsis [15–18].

In the current study, we enrolled a relatively large patient population (1480 neonates) and found that patients in the neonatal sepsis group had the highest level of NLR. To investigate the association of NLR levels with the presence of neonatal sepsis, subjects were divided into three groups according to NLR tertiles. Further analysis showed that the prevalence of neonatal sepsis showed a progressive increase from NLR tertile 1 to tertile 3. Multivariate analysis showed that NLR was an independent predictor of the presence of neonatal sepsis. Moreover, the ROC curve inferred an NLR cut-off value of 1.62 in predicting the presence of neonatal sepsis. Like NLR, PCT and hsCRP are also inflammatory markers, and many studies have demonstrated that elevated PCT and hsCRP were useful biomarkers for the diagnosis of neonatal sepsis, and elevated PCT and hsCRP were associated with the presence of neonatal sepsis [46–48]. In agreement with previous reports, our study also demonstrated that PCT and hsCRP are independently associated with the presence of neonatal sepsis (data not shown). Nonetheless, NLR was still an independent risk factor for neonatal sepsis when PCT and CRP were added to the multivariate regression model.

There are several limitations in our study. First, this is a cross-sectional and single-center study, which cannot predict future events and may have some inherent biases. Second, the diagnosis of neonatal sepsis was based on clinical features and was not confirmed by positive blood culture. Third, NLR was only measured at one time point. Serial measurements of NLR and the change in neonatal sepsis would provide more information on their relationship and would be useful for further exploring the dynamic correlation between them.

## 5. Conclusion

The current study demonstrated that there existed a relationship between NLR and the presence of neonatal sepsis. NLR level was higher in patients with neonatal sepsis and showed a gradual increase among three groups. Meanwhile, multivariate analysis showed that NLR was independently associated with the presence of neonatal sepsis. The findings highlight the potential value of NLR in predicting the risk of neonatal sepsis.

## Figures and Tables

**Table 1:** Baseline characteristics of control, infection, and sepsis group.

Variables	Controls (*n* = 188)	Infection (*n* = 555)	Sepsis (*n* = 737)	*P*
Age (days)	7.0 (5.0, 12.0)	7.0 (4.0, 12.0)	10.0 (5.0, 17.0)^bc^	<0.001
Male, *n* (%)	115 (61.2%)	318 (57.3%)	453 (61.5%)	0.295
Weight (kg)	3.3 ± 0.5	3.3 ± 0.5	3.2 ± 0.6^c^	0.014
Temperature (°C)	36.9 ± 0.3	36.9 ± 0.4	37.4 ± 0.8^bc^	<0.001
Respiratory (rate/minute)	45.4 ± 6.3	46.3 ± 7.6	49.8 ± 10.9^bc^	<0.001
Heart rate (bpm)	141.8 ± 12.6	142.4 ± 15.7	149.7 ± 19.6^bc^	<0.001
SBP (mm Hg)	76.9 ± 7.2	76.4 ± 6.9	76.1 ± 8.3	0.439
DBP (mm Hg)	47.1 ± 7.5	46.7 ± 7.1	45.9 ± 8.0	0.063
PCT (ng/mL)	0.11 (0.09, 0.18)	0.14 (0.10, 0.25)^a^	0.31 (0.14, 1.52)^bc^	<0.001
hsCRP (mg/L)	0.7 (0.7, 0.7)	0.7 (0.7, 0.7)	0.7 (0.7, 13.48)^bc^	<0.001
Biochemical parameters				
TBIL (*μ*mol/L)	306.4 (262.3, 357.7)	298.2 (230.9, 355.8)^a^	132.0 (45.4, 216.1)^bc^	<0.001
AST (U/L)	34.4 (28.5, 46.8)	37.9 (30.2, 51.0)^a^	38.5 (27.9, 55.2)	0.078
ALT (U/L)	24.3 (17.6, 30.3)	25.5 (20.0, 33.2)	28.5 (21.8, 37.8)^bc^	<0.001
TP (g/L)	57.5 ± 5.8	57.3 ± 6.7	54.1 ± 7.4^bc^	<0.001
ALB (g/L)	34.4 ± 3.7	33.8 ± 4.0	30.6 ± 4.9^bc^	<0.001
UREA (mmol/L)	2.2 (1.3, 2.9)	2.3 (1.5, 3.2)	3.1 (1.9, 4.4)^bc^	<0.001
CREA (*μ*mol/L)	49.6 (41.0, 57.9)	51.0 (42.0, 59.3)	47.3 (37.4, 63.1)^c^	0.057
UA (*μ*mol/L)	135.5 (104.9, 186.5)	137.9 (103.1, 182.5)	147.8 (109.9, 205.0)^bc^	0.003
Hematologic parameters				
WBC (10^9^ cells/L)	9.45 (7.92, 11.67)	10.03 (8.10, 12.39)	9.99 (7.27, 14.13)	0.253
Neutrophil count (10^9^ cells/L)	3.72 (2.82, 5.49)	4.29 (3.16, 6.07)^a^	5.07 (3.07, 8.58)^bc^	<0.001
Lymphocyte count (10^9^ cells/L)	4.10 (3.15, 4.86)	3.94 (2.95, 5.18)	3.34 (2.11, 4.78)^bc^	<0.001
NLR	0.92 (0.63, 1.45)	1.07 (0.75, 1.84)^a^	1.65 (0.85, 3.07)^bc^	<0.001

All values are presented as the mean ± SD or *n* (%) or as the median (interquartile range). SBP, systolic blood pressure; DBP, diastolic blood pressure; PCT, procalcitonin; TBIL, total bilirubin; AST, aspartate aminotransferase; ALT, alanine aminotransferase; TP, total protein; ALB, albumin; UREA, urea nitrogen; CREA, creatinine; UA, uric acid; WBC, white blood cell; hsCRP, high-sensitivity C-reactive protein; NLR, neutrophil-to-lymphocyte ratio. ^a^*P* < 0.05 for infection vs. control. ^b^*P* < 0.05 for sepsis vs. control. ^c^*P* < 0.05 for sepsis vs. infection.

**Table 2:** Clinical and demographic characteristics according to NLR tertiles.

Variables	First tertile (< 0.91) (*n* = 493)	First tertile (0.91-1.88) (*n* = 493)	Third tertile (> 1.88) (*n* = 494)	*P*
Age (days)	11.0 (7.0, 19.0)	8.0 (4.0, 13.0)^a^	5.0 (3.0, 10.0)^bc^	<0.001
Male, *n* (%)	298 (60.4%)	290 (58.8%)	298 (60.3%)	0.846
PCT (ng/mL)	0.11 (0.09, 0.18)	0.16 (0.10, 0.31)^a^	0.50 (0.21, 2.86)^bc^	<0.001
hsCRP (mg/L)	0.7 (0.7, 0.7)	0.7 (0.7, 0.7)	0.7 (0.7, 15.53)^bc^	<0.001
Biochemical parameters				
TBIL (*μ*mol/L)	258.9 (93.7, 335.3)	260.8 (126.2, 330.0)	191.2 (111.1, 278.3)^bc^	<0.001
AST (U/L)	37.0 (29.1, 51.7)	36.1 (28.3, 48.3)	40.3 (29.3, 57.1)^c^	0.009
ALT (U/L)	26.7 (21.4, 35.7)	25.5 (19.3, 33.1)^a^	27.8 (21.0, 36.6)^c^	0.005
TP (g/L)	57.0 ± 6.3	56.1 ± 7.6	54.0 ± 7.1^bc^	<0.001
ALB (g/L)	33.4 ± 4.4	32.6 ± 4.6^a^	30.7 ± 4.9^bc^	<0.001
UREA (mmol/L)	2.3 (1.4, 3.2)	2.4 (1.6, 3.9)^a^	3.1 (2.1, 4.7)^bc^	<0.001
CREA (*μ*mol/L)	44.8 (37.2, 52.5)	48.8 (40.0, 58.1)^a^	57.0 (43.1, 75.7)^bc^	<0.001
UA (*μ*mol/L)	130.3 (100.6, 159.9)	138.9 (104.5, 185.4)^a^	170.1 (118.9, 253.5)^bc^	<0.001
Clinical data				
Control, *n* (%)	91 (18.5%)	65 (13.2%)	32 (6.5%)	<0.001
Infection	197 (40.0%)	223 (45.2%)	135 (27.3.0%)^bc^	<0.001
Sepsis	205 (41.6%)	205 (41.6%)	327 (66.2%)^bc^	<0.001

Abbreviations as in Table 1. ^b^*P* < 0.05 for sepsis vs. control. ^c^*P* < 0.05 for sepsis vs. infection.

**Table 3:** Correlations between NLR and clinical parameters.

Variables	*r*	*P*
Age (day)	-0.378	< 0.001
PCT (ng/mL)	0.531	< 0.001
hsCRP (mg/L)	0.255	< 0.001
TBIL (*μ*mol/L)	-0.111	< 0.001
AST (U/L)	0.050	0.053
ALT (U/L)	0.023	0.385
TP (g/L)	-0.187	< 0.001
ALB (g/L)	-0.249	< 0.001
UREA (mmol/L)	0.251	< 0.001
CREA (*μ*mol/L)	0.294	< 0.001
UA (*μ*mol/L)	0.232	< 0.001

Abbreviations as in Table 1.

**Table 4:** Multivariate logistic regression analysis for prediction of neonatal sepsis.

Model	Variables	OR (95% CI)	*P*
Model 1	NLR	1.450 (1.340-1.569)	< 0.001
	NLR tertiles		
	Tertile 1	1	
	Tertile 2	1.000 (0.776-1.288)	1.000
	Tertile 3	2.751 (2.124–3.562)	< 0.001

Model 2	NLR	1.445 (1.301–1.604)	< 0.001
	NLR tertiles		
	Tertile 1	1	
	Tertile 2	1.116 (0.804-1.550)	0.511
	Tertile 3	2.796 (1.941–4.029)	< 0.001

Model 3	NLR	1.331 (1.190–1.489)	< 0.001
	NLR tertiles		
	Tertile 1	1	
	Tertile 2	0.987 (0.707-1.378)	0.938
	Tertile 3	2.039 (1.395–2.980)	< 0.001

Abbreviations as in Table 1. Model 1: unadjusted. Model 2: adjusted for age, heart rate, respiratory rate, weight, TP, ALB, CREA, and TBIL. Model 3: adjusted for age, heart rate, respiratory rate, weight, TP, ALB, CREA, TBIL, PCT, and hsCRP.

## Data Availability

The data used to support the findings of this study are available from the corresponding author upon request.
